# Impact of Tetrapeptide-FSEY on Oxidative and Physical Stability of Hazelnut Oil-In-Water Emulsion

**DOI:** 10.3390/foods10061400

**Published:** 2021-06-17

**Authors:** Chenshan Shi, Miaomiao Liu, Qinghua Ma, Tiantian Zhao, Lisong Liang, Bolin Zhang

**Affiliations:** 1 Beijing Key Laboratory of Forest Food Processing and Safety, College of Biological Sciences and Technology, Beijing Forestry University, Beijing 100083, China; shichenshan@163.com (C.S.); 17853555780@163.com (M.L.); 2 Key Laboratory of Tree breeding and Cultivation of the State Forestry and Grassland Administration, Research Institute of Forestry, Chinese Academy of Forestry, Beijing 100091, China; mqhmary@caf.ac.cn (Q.M.); zhaotian1984@163.com (T.Z.); 3 State Key Laboratory of Tree Genetics and Breeding, Beijing 100091, China; 4 Hazelnut Engineering and Technical Research Center of the State Forestry and Grassland Administration, Beijing 100091, China; 5 National Innovation Alliance of Hazelnut Industry, Beijing 100091, China

**Keywords:** antioxidant, FSEY, oil-in-water emulsion, lipid oxidation, physical stabilities

## Abstract

This study investigates the antioxidant behaviors of a hazelnut tetrapeptide, FSEY (Phe-Ser-Glu-Tyr), in an oil-in-water emulsion. The emulsion was prepared with stripped hazelnut oil at a ratio of 10%. O/W emulsions, both with and without antioxidants (FSEY and TBHQ), were incubated at 37 °C. The chemical stabilities, including those of free radicals and primary and secondary oxidation productions, along with the physical stabilities, which include particle size, zeta-potential, color, pH, and ΔBS, were analyzed. Consequently, FSEY displayed excellent antioxidant behaviors in the test system by scavenging free lipid radicals. Both primary and secondary oxidation products were significantly lower in the FSEY groups. Furthermore, FSEY assisted in stabilizing the physical structure of the emulsion. This antioxidant could inhibit the increase in particle size, prevent the formation of creaming, and stabilize the original color and pH of the emulsion. Consequently, FSEY may be an effective antioxidant additive to use in emulsion systems.

## 1. Introduction

The oxidative rancidity of edible oils with high unsaturated fatty acid contents is a major concern in the food industry, because it is directly related to nutrition, flavor, safety, and storage problems [1]. Moreover, the susceptibility of oils to oxidation is also a major cause of quality deterioration in emulsions [2]. As an essential aspect of food system, such as milk, infant formula, salad dressing, mayonnaise, cream, etc., oil-in-water emulsions consist of three different components: water, oil, and emulsifiers. Although the oxidation of unsaturated fatty acids is responsible for emulsion deterioration, the mechanism of lipid oxidation in oil-in-water emulsions is more complex than that in bulk oils [1]. This oxidation occurs mostly at the water–oil interface rather than the air–water interface [3]. As such, the free radical chain reaction, shown in the following, occurs in the interfacial region, which is the contact region between the oil phase and aqueous phase. Oxidation products, including lipid hydroperoxides as primary products and aldehydes and ketones as secondary products, are generated at the interface [4]. Notably, water-soluble ingredients, such as metals in the aqueous phase and emulsifiers at the interface, profoundly influence the quality of food emulsions during processing and storage. For these reasons, the oxidation of an oil-in-water emulsion is much faster than oil in its bulk form [5]. However, lipid oxidation can be controlled by a variety of technologies, such as different packaging and various storage conditions; notably, the addition of antioxidants is the most important control method in the food industry, and especially of antioxidants obtained from plants.


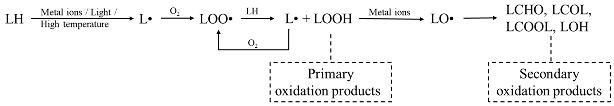


In addition to changes in chemical composition, the physical stability also changes during storage. The main mechanisms governing emulsions’ instabilities are drainage- and gravity-driven separations, namely, creaming, sedimentation, flocculation, coalescence, and Ostwald ripening. Flocculation means that two or more droplets attract each other to form flocs, but maintain their inherent properties, coalescence in the merging of two droplets into a single droplet; Ostwald ripening then results in a mass transfer from small to large droplets, eventually leading to macroscopic stratification [6,7]. Destabilization processes are interconnected and influence each other. Flocculation tends to make the droplet population more compact and consequently may accelerate the coalescence processes. Coalescence and Ostwald ripening modify the drop size distribution, and an increase in the average droplet size eventually destabilizes the emulsion. Additionally, increasing the temperature will improve particle movement, resulting in flocculation, coalescence, and Ostwald ripening. Phytic acid [8], EDTA (ethylenediaminetetraacetic acid) [9] and catechin [10] can help to stabilize both the physical and chemical stabilities of emulsions. Due to the lack of acceptable consumers and effective metal chelators, the utilization of free radical scavenging antioxidants to control lipid oxidation in oil-in-water emulsions has become a trend [11].

In summary, the quality and shelf life of emulsions are determined by their oxidative and physical stabilities, which mainly include their ability to resist changes in chemical structure and spatial distribution over time [3]. As pointed out, synthetic antioxidants, such as BHA, BHT, and TBHQ, and natural products, such as polyphenols [3], phytic acid [8], catechin [10], phospholipids, and tocopherols [12], can act as antioxidants in oil-in-water emulsions; additionally, peptides can act as both antioxidants and emulsifiers in this system [13,14]. These antioxidants can slow the accumulation of primary and secondary lipid oxidation products via reactive oxygen species scavenging, free radical scavenging, and metal chelating. However, peptides with defined amino acid sequences have not been reported as additives in emulsions.

FSEY (Phe-Ser-Glu-Tyr) is a tetrapeptide derived from hazelnut hydrolysates. Previous research has demonstrated that FSEY is an excellent antioxidant against linoleic acid (unpublished data). In this study, FSEY is added to a hazelnut oil-in-water emulsion system to evaluate antioxidant behaviors. The chemical stabilities, including free radicals and primary and secondary oxidation products, along with the physical stabilities, which include the particle size, zeta potential, color, pH, and dispersion stability, are used as evaluation indices.

## 2. Materials and Methods

### 2.1. Materials

A hazelnut tetrapeptide, FSEY (Phe-Ser-Glu-Tyr. Mw = 544.57), was synthesized using a solid-phase peptide synthesis method by Zhejiang Ontores Biotech Co., Ltd. (Hangzhou, Zhejiang, China) with a purity of 99.14%. The hazelnut oil was produced by a screw rod press (Model CZR309, Wanbiao Co., Ltd., Guangzhou, China). PBN (N-tert-butyl-α-phenylnitrone) was purchased from Sigma Aldrich (Zwijndrecht, The Netherlands). Nile red was purchased from Shanghai EKEAR Bio@Tech Co. Ltd. (Shanghai, China). All other analytical-grade reagents were purchased from local suppliers.

### 2.2. Preparation of Stripped Hazelnut Oil

**Figure 1:**
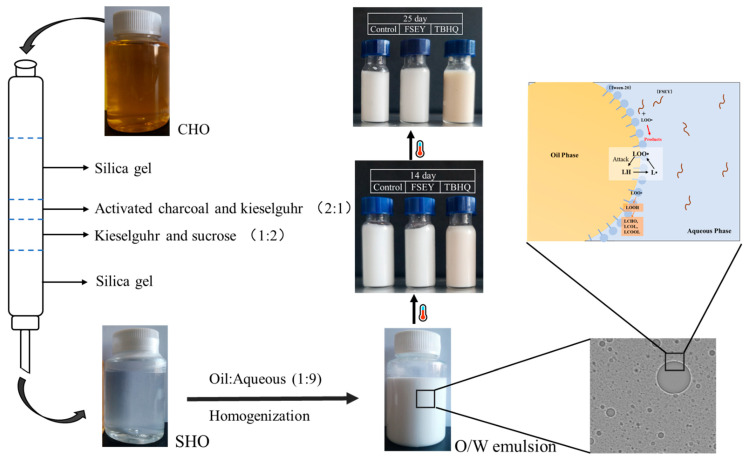
Procedures for preparing purified oil and its emulsion.

To study the antioxidant behavior of FSEY without any other antioxidants in oil, crude hazelnut oil (CHO) was stripped according to the methods of Samdani et al. [12]. As shown in Figure 1, four layers were packed into a column (2.4 cm internal diameter × 40 cm height), including 37.5 g of silica gel (activated at 110 °C for 4 h), 6.3 g of activated charcoal and kieselguhr (2:1), 19 g of kieselguhr and sucrose (1:2), and another 37.5 g of silica gel. Crude oil (50 g) was mixed with 50 mL of hexane, and the triacylglycerol fraction was eluted using hexane. The solvent containing oil was then removed in a fume hood. The stripped hazelnut oil (SHO) was transparent and free of tocopherol, sterol, and β-carotene. SHO was stored in the dark at −20 °C until use.

### 2.3. Emulsion Preparation and Storage Conditions

The oil-in-water emulsions were prepared as previously reported with some modifications [8]. Emulsions were fabricated by homogenizing 90% (*w*/*w*) aqueous phase (5 mM phosphate buffer, pH 7.0) and 10% (*w*/*w*) oil phase. Tween-20 was used as the emulsifier at a 0.5% (*w*/*w*) ratio. Sodium azide (NaN_3_, 0.02% (*w*/*w*)) was used as an antibacterial agent. Emulsions were prepared in two successive steps. A pre-emulsion was obtained by high-shear mixing (D1000 hand-held homogenizer, Benchmark, Angleton, TX, USA) at 30,000 rpm for 2 min. The high-pressure homogenization of the pre-emulsion was then achieved using a nano-homogenize machine (ATS Engineering Inc., Brampton, ON, Canada) by applying three homogenization cycles at 700 bar. The resulting emulsion was then used to prepare emulsions containing different levels of FSEY (0.01%, 0.015%, 0.02% *w*/*w*) or TBHQ (tertiary butylhydroquinone, 0.02% *w*/*w*). All the samples were then incubated in the dark for 25 days (37 °C). The physical and chemical indices were analyzed at selected times.

### 2.4. Chemical Indices: Oxidation Level

#### 2.4.1. Determination of the LOOH Value

During incubation, the concentrations of primary (lipid hydroperoxide (LOOH) and conjugated diene (CD)) and secondary (*p*-Av) lipid oxidation products were monitored to assess the oxidative stability of the emulsions.

According to the method of Shi [15], 0.3 mL of emulsion was added to 1.5 mL of isooctane and 2-propanol (3:1 *v*/*v*). The mixed solution was vortexed three times for 10 s each. Then, the solution was centrifuged at 1000× *g* for 2 min. Next, 0.2 mL of supernatant was reacted with 15 μL of ammonium thiocyanate (3.94 M) and 15 μL of a ferrous solution (obtained from the supernatant of a mixture of 1 mL of 0.132 M BaCl_2_ and 1 mL of 0.144 M FeSO_4_), followed by a dilution with 2.8 mL of mixed methanol and 1-butanol (2:1, *v*/*v*). Absorption was measured at 510 nm after 20 min of reaction in darkness. The lipid hydroperoxide concentrations were determined using a standard curve of cumene hydroperoxides.

#### 2.4.2. Determination of the CD Value

The formation of conjugated dienes (CDs) was monitored by UV-Vis spectroscopy according to Pascual et al. with some modifications [16]. In brief, 25 µL of the emulsion was diluted to 5 mL with ethanol and then vortexed three times. The absorbance was measured at 233 nm. The CD content was calculated by Equation (1).

(1) CD=A233nm[oil]×ε×L

[oil], the concentration of oil, kg/L;
L, optical path of colorimetric ware, 1 cm;
ε, molar absorptivity of conjugated dienes, 27,000 M^−1^cm^−1^.

#### 2.4.3. Determination of the *p*-Anisidine Values

The secondary products were expressed by the anisidine value (*p*-Av) [17,18]. Adaptations were made to destabilize the emulsions: 1 mL of ethanol was added to 1 mL of emulsion, followed by 2 mL of iso-octane. Ethanol was used to destabilize the emulsion so that the oil was then exposed and free to disperse in the isooctane. The reaction system was vortexed for ten seconds and centrifuged for 20 min at 4000× *g*. Then, 2 mL of the transparent upper solvent layer was mixed with 1 mL of *p*-anisidine (prepared by dissolving 0.25 g of anisidine in 100 mL of acetic acid). The absorbance was measured at a wavelength of 350 nm.

(2) p−Av=100×Q×V (1.2A1−A2)m

Q, defined value of anisidine, 0.01 g/mL;
V, volume of the sample, mL;
1.2, correction factor of 1 mL anisidine–acetic acid solution;
M, weight of the oil, g;
A_1_, absorbance of the samples;
A_2_, absorbance of isooctane + *p*-anisidine solution.

#### 2.4.4. Determination of the LOO•

To detect lipid free radicals, PBN was added into the samples with a final concentration of 50 mM before incubation [19]. All the samples were incubated at 37 °C. Every single part was retrieved on days 14 and 25. The following parameters were used in the ESR (electron spin resonance) measurements: center field, 322.500 mT; sweep time, 2.0 min; modulation width, 1.0 × 0.1 mT; microwave power, 0.99800 mW; modulation frequency, 100 kHz. The radical intensity was defined as the PBN-radical adduct signal and the signal of the Mn (II) marker [20] by ESR spectroscopy (JEOL FA-200, JEOL Ltd., Tokyo, Japan).

### 2.5. Physical Indices

#### 2.5.1. Particle Size and Charge Measurements

The particle-size distributions of the emulsions were determined with a laser particle size analyzer (LS 13320, Beckman Coulter Inc., Brea, CA, USA). Particle sizes are reported as the volume mean diameter (D_4,3_). The electrical charge of the oil droplet was evaluated by measuring the zeta potential (mV) by a zeta potentiometer (Brookhaven Zeta PALS, Brookhaven Instruments Inc., Holtsville, NY, USA). Before zeta potential analysis, the emulsion was diluted 5 times with PB. The 1.3 mL diluted samples were removed to sample cells. The following parameters were used: measurements, 3; starting temperature, 25.0 °C; liquid, water; zeta potential model, Huckel.

#### 2.5.2. Microstructural Analysis

Microstructures were observed using laser scanning confocal microscopy (LSCM) with a 60× objective lens (Zeiss LSM710 3-channel, Zeiss, Germany). For the fluorescence measurements, 200 μL of sample was pre-stained with 15 μL of Nile red solution (0.02%, dissolved in ethanol) to dye the lipid phase for 2 h in the dark. Then, 3 μL of stained emulsion was loaded on a slide and covered with a coverslip. The excitation wavelength used for the Nile red dye was 515 to 560 nm. In addition, the sizes of the oil droplets in the pictures were also analyzed by the open software ImageJ.

#### 2.5.3. Color Measurement

The change in color of the emulsions was evaluated using a colorimeter (CS-580, Hangzhou Caipu Technology Co., Ltd., Hangzhou, Zhejiang, China). The overall colors were presented with a white standard plate (L* = 91.74, a* = −0.54, b* = 0.99) as the background. The L (lightness), a (redness) and b (yellowness) values for each sample were measured, and the mean values were recorded [21]. The total color difference, ΔE, was calculated according to Equation (3).

(3) $$\Delta E=\sqrt{{\left(L^{*}-L\right)}^{2}+{\left(a^{*}-a\right)}^{2}+{\left(b^{*}-b\right)}^{2}}$$

#### 2.5.4. Turbiscan Stability Index

The physical stability of emulsions was monitored through a Turbiscan optical analyzer (TURBISCAN AGS, Formulaction, France). Samples were transferred into transparent flat-bottomed test tubes and measured over 24 h. Then, 97 scans of each sample were collected with 15 min interval between each scan. The monitoring was performed at 25 °C and 60 °C. The stability analysis was performed as a variation of backscattering (BS) profiles as a function of time at the bottom, middle, and top layers of the samples, and was then exported as ΔBS by Towersoft 2.0. Stabilities were also calculated as the Turbiscan stability index (TSI).

### 2.6. Antioxidant Capacity of FSEY at High Temperatures

To evaluate the antioxidant ability of FSEY in O/W at high temperatures, the emulsions were stored at 60 °C and 120 °C for some time. LOOH was then measured as the antioxidant index.

### 2.7. Antioxidant Capacity of FSEY against Other Radicals

DPPH radicals and ABTS radicals were used to evaluate the capability of FSEY against other radicals. The radical scavenging activity of the sample was evaluated according to a previous method with some modifications [22]. Briefly, FSEY–water solutions were mixed with 0.1 mmoL/L DPPH (prepared in ethanol) at a ratio of 1:1 (*v*/*v*). The absorbance value was detected at 517 nm after incubation in the dark for 30 min. The ABTS radical was generated by mixing the same volumes of 7 mM ABTS and 2.45 mM potassium persulfate, and kept in the dark at room temperature for 12 h. The absorbance of ABTS at 734 nm was controlled to 0.70 ± 0.02 by diluting with 5 mM phosphate buffer (pH 7.4). Next, 0.1 mL samples were added to 3.9 mL of ABTS radical solution. The absorbance value was detected at 734 nm after incubation in the dark for 100 min. Deionized water instead of sample was used as a negative control (blank), and GSH was used as a positive control. The results are expressed as GSH equivalents. DPPH and ABTS scavenging activities were calculated by Equation (4).

(4) Scavenging activity (%)=(1−AsampleAblank)×100

### 2.8. Distribution of FSEY in an Emulsion

The distribution of FSEY in a binary oil–water system (without Tween-20) was determined by high-performance liquid chromatography (HPLC) analysis on a SHIMADZU system (MODEL: LC-20A, SHIMADZU, Kyoto, Japan) equipped with an SPD-M20A detector. InertSustain C18 (5 µm, 4.6 × 150 mm) was used as the column, with the following parameters: a flow rate of 1.0 mL/min, detection at 220 nm, and an eluent system of A = 0.1% TFA in H_2_O, B = acetonitrile. The following gradient was applied: 10–30% B over 20 min. FSEY was dissolved in the binary oil–water system at concentrations of 0.1, 0.15 and 0.2 mg/mL. After becoming stable overnight, the emulsions were centrifuged at 3000× *g* for 30 min. The water phases were filtered through a 0.45 μm membrane filter for HPLC detection.

### 2.9. Prediction of the Toxicological Properties of FSEY

We predicted the toxicological properties of FSEY via the widely used commercial toxicity predictor software TOPKAT (Toxicity Prediction by Komputer Assisted Technology).

### 2.10. Statistical Analysis

All experiments were performed in duplicate or triplicate and reported as the mean standard deviation. IBM SPSS 26.0 software was used for the statistical analysis between groups. The data are expressed as the mean ± standard deviation.

## 3. Results and Discussion

### 3.1. Lipid Oxidative Stability

**Figure 2:**
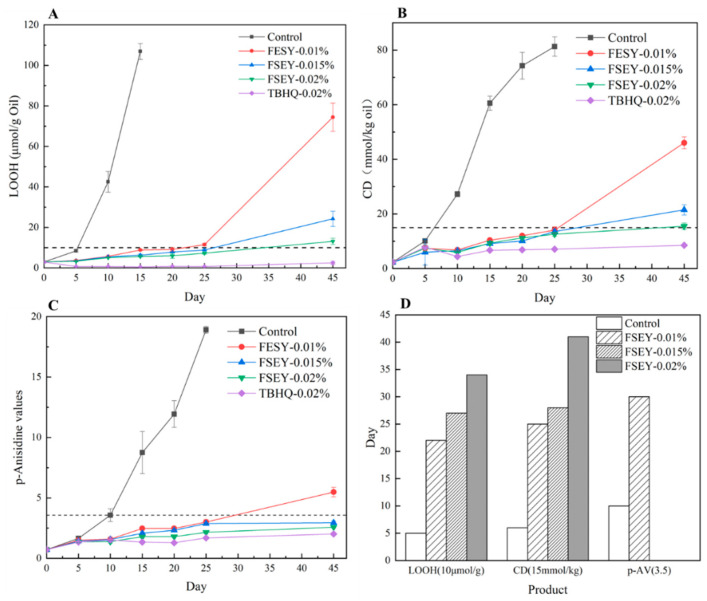
(**A**–**C**) Changes in primary (LOOH and CD) and secondary (*p*-Av) lipid oxidation product values stored at 37 °C for 25 days. Values are expressed as mean ± SD (*n* = 3). (**D**) The replot of Figure 2A–C showing the effect of FSEY at different levels on time required to increase the LOOH, CD, and *p*-AV contents to 10 μg/mL, 15 mmol/kg, and 3.5, respectively.

Protein hydrolysates have been determined to be antioxidants that can slow lipid auto-oxidation [23]. Due to the oil-insoluble nature of FSEY, an oil-in-water emulsion was prepared. Figure 2 shows the primary (LOOH and CD) and secondary (*p*-Av) lipid oxidation product values as evaluations of the emulsion oxidative stabilities.

Lipid hydroperoxides, LOOH, are prominent non-radical intermediates of oil peroxidation [2] that are widely used to detect the oxidation of oil in emulsion. During the storage period, the LOOH value exhibited an increasing tendency. As Figure 2A,D show, the LOOH value of the control sample was nearly 10 μmol/g oil on day 5, and this increased quickly during storage. In comparison, the LOOH value of the emulsion with 0.01% FSEY reached the same value on day 22. Emulsions with 0.015% and 0.02% FSEY reached this value after approximately 27 and 34 days, respectively. Nevertheless, emulsions with the synthetic antioxidant, TBHQ, showed the lowest LOOH values throughout the storage period. It has been reported that protein hydrolysates acquired from rice [14], soya [24], quinoa, and amaranth [25] exert a strong protective effect against the oxidative deterioration of emulsions by inhibiting the formation of hydroperoxides. Amino acids (e.g., tryptophan, methionine, and tyrosine) are reported to be involved in the antioxidant activity of emulsion systems by scavenging free radicals [26].

CD values are indicators of free radical production, reflecting the extent of lipid auto-oxidation at an early stage [2]. The appearance of CD in oxidized lipids is attributed to an electronic shift due to a radical attack of methylene groups that separate double bonds, which are mainly found in PUFA [1]. During the storage period, conjugated diene structures formed. As Figure 2B shows, the CD values show an increasing trend during the storage period. The value reached 15 mmol/kg oil without any antioxidants after approximately 6 days. However, for the 0.01%-FSEY, 0.015%-FSEY, and 0.02%-FSEY groups, the time extended to nearly 25, 28, and 41 days, respectively. Therefore, FSEY significantly inhibited conjugated diene formation. This phenomenon corresponded to the LOOH values. In summary, FSEY could inhibit the formation of primary oxidation products in an oil-in-water emulsion.

The progress of lipid oxidation in the emulsions was also monitored via the *p*-anisidine value. As the primary product, hydroperoxide is unstable and easily breaks up into secondary compounds, such as volatile aldehydes (hexanal), through complex fragmentation and interaction, leaving behind a non-volatile portion that remains a part of the lipid molecule [2]. These non-volatile products can be measured by their reaction with anisidine. From Figure 2C,D, the *p*-anisidine value increased over 45 days, and the value of the control sample was 3.57 ± 0.53 on day 10. Additionally, the presence of 0.01% FSEY could extend the time to reach this value to nearly 30 days. Notably, the 0.015% FSEY, 0.02% FSEY and 0.02% TBHQ groups did not reach this value during the storage period.

According to the contents of lipid hydroperoxides, conjugated diene values, and anisidine values, FSEY showed antioxidant activities during auto-oxidation in a concentration-dependent manner. In the incubation stage, the oxidative progression to rancidity in oil occurred rapidly. Both TBHQ and FSEY displayed notable antioxidant capacity. At the same concentration of 0.02%, FSEY was similar in capacity to TBHQ. These results suggest that as a natural product, peptides could be a good additive in emulsion systems.

### 3.2. Lipid Free Radicals

**Figure 3:**
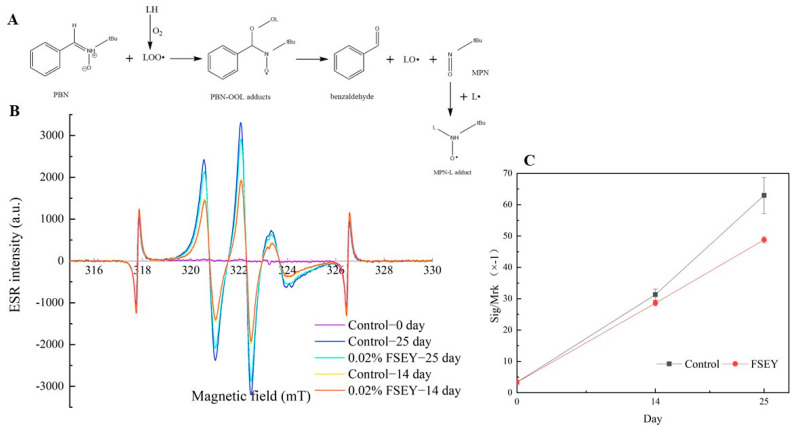
(**A**) Mechanisms of the interaction of PBN with lipid radicals and the fate of its products in emulsions. (**B**) Evolution of peak-to-peak amplitude of spin adducts after heated storage (37 °C for 25 days). (**C**) Evolution of the radical relative intensity (signal/marker) during the accelerated oxidative process. Values are expressed as mean ± SD (*n* = 2).

ESR is a useful technique for the detection of free radicals. LOO• are formed during lipid oxidation. Free radicals are highly reactive and acquire steady-state concentrations, which are difficult to directly detect by ESR. As shown in Figure 3A, PBN, as spin-traps, react with LOO• and form PBN-OOL. This product rapidly decomposes to alkoxy-radicals (LO•), benzaldehyde and 2-methyl-2-nitrosopropane (MNP). Then, MNP reacts with L• radicals to form MNP-L adducts that can actually be measured with ESR [27]. Figure 3B shows the signal evolution of emulsions with and without FSEY at days 0, 14 and 25. ESR signals with three hyperfine lines were clearly distinguishable. Notably, ESR spectroscopy did not identify MNP-L at day 0. Nevertheless, the radical signals increased during the storage period, and FSEY showed an inhibitory effect. We also used the ratio of signal/marker (Sig/Mrk) as an indicator of radical contents. As shown in Figure 3B, FSEY inhibited the accumulation of MNP-L by scavenging lipid free radicals.

**Figure 4:**
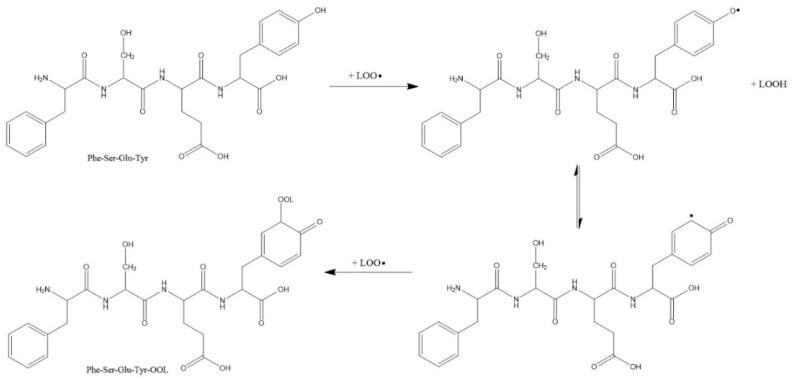
The potential mechanism of the FSEY scavenging peroxy-radicals (LOO•).

We found that the -Tyr residue is responsible for the antioxidant activity of peptides (unpublished data). Based on the study of Wu [28], peptide-FSEY, containing hydrogen donors (such as Try), acidic amino acids (such as Glu) and hydrophobic amino acids (such as Phe), could be determined to be an antioxidant that complexes free radicals, thereby delaying or inhibiting the initiation stages or interrupting the propagation stages of lipid oxidation; this mechanism is similar to that of phenolic compounds [29]. It has also been reported that the phenolic hydroxyl group in Tyr is relatively easy to oxidize [30]. In addition, with longer lifetimes than peroxy-radicals, much more stable phenoxy radicals inhibit any reverse reaction or the propagation of the radical-mediated peroxiding chain reaction [31]. According to the reaction mechanism of ATVY against ABTS•^+^ [22], peroxy-radicals may be linked to the ortho-position of the phenoxy in Tyr by covalent bonds. The Tyr- residue could also be a hydrogen donor in this system. The possible mechanism is thus shown below (Figure 4).

The GRAVY (grand average of hydropathy) of FSEY was calculated by a gravy-calculator (online, http://www.gravy-calculator.de/). The value is −0.7, which stands for weakly hydrophilic or amphipathic. As the interfacial region is responsible for the development of lipid oxidation, antioxidants with suitable hydropathy show excellent activity at the interface. We analyzed the GRAVY values of peptides derived from the BIOPEP database (http://www.uwm.edu.pl/biochemia/, accessed on 10 June 2020) with an inhibitory effect on linoleic acid oxidation. The values varied from −3.2 to 1.52, while −1~0 accounted for a significant proportion, as shown in Table S1 and Figure S1. From this point of view, FSEY could be a good antioxidant in emulsions.

Antioxidant behaviors in bulk oils or in oil-in-water emulsions have been discussed for many years. In 1980, Porter proposed the antioxidant polar paradox, which stated that polar antioxidants work best in bulk oils, while nonpolar antioxidants work best in oil-in-water emulsions. Furthermore, Porter suggested that nonpolar antioxidants only needed to be retained at the lipid droplet interface because oxidative action occurred at the surface [32]. Apart from the physical location, antioxidants impact lipid oxidation kinetics in multiple ways. Hydrophilic antioxidants, such as ascorbic and gallic acids, are vigorous free radical scavengers that may be prooxidative in emulsions; in contrast, hydrophobic antioxidants, such as tocopherol, accelerate lipid oxidation at higher concentrations [5]. HPLC was used to analyze the distribution of the antioxidant in a binary oil–water system. The results showed that 97.52% ± 2.24 of FSEY dissolved in the aqueous phase, which means that the free radical scavenging reaction occurred at the interface, as shown in Figure 1. Notably, unlike tocopherol, the peptide did not show any prooxidative activity.

### 3.3. Particle Size, Appearance and Zeta Potential (mV)

**Figure 5:**
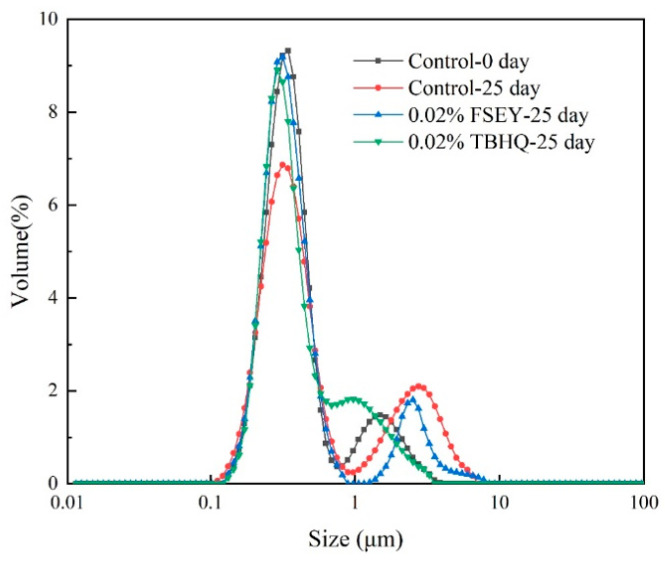
Particle size distribution.

Table 1 and Figure 5 show the mean size values and size distribution. The initial mean droplet diameters of the emulsion were 0.52 μm for d_4,3_. The addition of 0.02% FSEY or TBHQ did not affect particle size. During incubation, the particle size changed over time. After incubated for 25 days, the value increased to 0.84 μm. FSEY-containing emulsions showed higher particle stability than the control with a size of 0.69 μm. However, TBHQ could stabilize the particle size better. As Figure 5 shows, the size distribution changes after incubation. The volume (%) showed a decreasing trend between 0.1 and 1 μm after storage. Moreover, the percentage of droplets between 1 and 10 μm increased. FSEY and TBHQ could weaken this phenomenon. Therefore, particle size could indicate possible destabilization phenomena because, during incubation, tiny oil droplets gathered into large drops.

**Figure 6:**
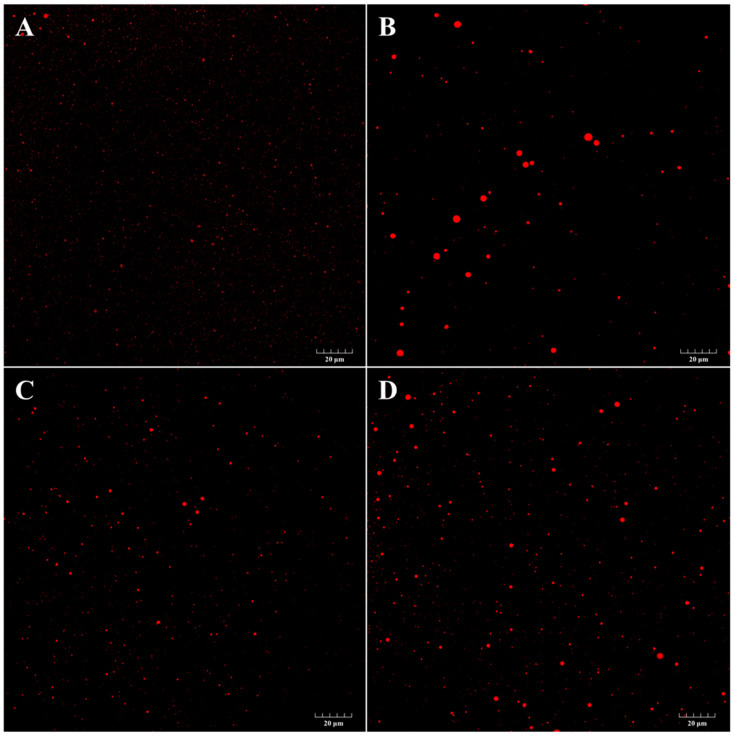
Laser scanning confocal micrographs of emulsions. The oil phase (red) was stained with Nile red excited at 541 nm. The scale bar is 20 μm. (**A**) Control-0 days, (**B**) Control-25 days, (**C**) FSEY-25 days, and (**D**) TBHQ-25 days.

Changes in the particle size could also be observed by LSCM, and the results were consistent with the size analysis. Figure 6 pictures oil drops evenly distributed in water. After staining with Nile red and excitation at 541 nm, the oil drops appeared with a red color. The distributions of droplets in the LSCM pictures were analyzed by ImageJ, and are shown in the Supplementary Material (Figure S2). For fresh emulsions, very fine lipid droplets were evenly distributed in the aqueous phase (black, A) with the maximum size of no more than 2.12 μm. Compared with the fresh emulsion, the oil droplet sizes clearly increased after incubation for 25 days. From Figure 6 and Figure S2, emulsions without any antioxidants showed the largest particle size. Within the field of view, some droplets in Figure 6B displayed larger sizes than 4 μm. The maximum size was nearly 4.74 μm. However, emulsions containing FSEY (C) and TBHQ (D) showed smaller diameters, with maximum sizes of no more than 2.82 and 3.88 μm, respectively. This phenomenon may account for their ability to stabilize the physical structure of emulsions. Due to their small particle size, nano-emulsion and submicron-sized emulsion (with particle sizes ranging from 50 nm to 200 nm and from 200 nm to 1000 nm) were resistant to Ostwald ripening [33]. Notably, we have not observed this phenomenon in LSCM. To conclude, the increase in particle size was due to flocculation and coalescence processes. The FESY-mediated inhibition of these processes may be attributed to its ability to sequester oil drops. The presence of the antioxidants at the interface phase may enhance the repulsive forces between oil droplets, thereby improving their stability [34]. However, to our dismay, the tetrapeptide could not be stained by Nile blue or FITC (fluorescein isothiocyanate); thus, it cannot be observed by LSCM. Here, the mean particle diameter of the TBHQ group measured by a laser particle size analyzer was lower than that of the FSEY group, which was a little inconsistent with the particle aggregation observed in the confocal microscopy images. This phenomenon might be explained by the different sample pre-treatments for the two analytical techniques.

Zeta potential values are another index to evaluate physical stability, indicating the adsorption of species at the oil–water interface [9,10]. It has been reported that emulsions with Tween-20 and Tween-80 as emulsifiers will result in a negative charge [12]. The immediate determination of the ζ-potential at day 0 without any additives was −51.03, while the addition of FSEY or TBHQ improved the value to −42.38 and −49.43, respectively. This effect may be due to the physical binding of FSEY/TBHQ to the surfaces of the adsorbed emulsifier. Our observations are in good agreement with those reported by Yi et al. [34] and Cheng et al. [35], who found that the addition of anthocyanins and flaxseed polyphenols into oil-in-water emulsions resulted in a reduction in the absolute value of ζ-potential. Furthermore, changes in the surface potential during storage are indicative of changes in the interfacial composition. In the case of hazelnut oil emulsions after 25 days of storage, the absolute values of zeta potentials showed a slightly increasing trend (Table 1). The ζ-potentials of the control group, the FSEY group, and the TBHQ group decreased to −55.01, −46.51, and −50.09, respectively. This result may be ascribed to the anionic reaction products, such as free fatty acids formed by lipid oxidation or hydrolysis, as well as to the loss of cationic reactants, such as amino groups, on the peptide during storage [34]. The formation of free fatty acids led to a decrease in pH, as listed in Table 1.

### 3.4. Color Changes of the Emulsions

The colors of the emulsions were evaluated. As shown in Figure 1, compared to other groups, emulsions with TBHQ turned yellow and red after a period of storage at 37 °C, which may be attributed to the formation of quinones from phenols. These color parameters were analyzed and are shown in Table 2. The lightness value (L) is often determined by the scattering effect, while the redness (a) and yellowness (b) are dependent on absorption [36]. Unlike the FSEY group, the control group and TBHQ groups showed a significant reduction in their lightness values (L). The changes from bright white to grey indicate a decrease in the L value, and an increase in the particle size distribution, owing to the reduced light scattering effect [36]. Here, the decrease in the L values of the control group corresponded to the increase in particle size, which is in good agreement with the particle size analysis and those analyses reported by others [36,37]. Although the L values in the TBHQ group significantly decreased, we did not find an increase in particle size via the laser particle size analyzer and LSCM. Hence, the reason for the decrease in L values needs further analysis. The redness (a) and yellowness (b) values showed slightly decreasing and increasing trends in the control and FSEY groups, respectively. However, there were noticeable increases in a and b values in the TBHQ group, which can be identified in Figure 1. In addition, the total color differences (ΔE) suggest that TBHQ affected the emulsion color during incubation, while FSEY stabilized the initial color.

### 3.5. Stabilities of the Emulsions

Oil-in-water emulsions tend to destabilize, which can be distinguished in real-time by detecting the optical properties of the emulsions with a Turbiscan instrument [38]. Variations in the backscatter intensity (BS) [39] and Turbiscan stability index (TSI) [40] provide qualitative indications of changes in droplet distribution during destabilization, which takes into account destabilization phenomena. To evaluate the effects of heat treatment and antioxidants on emulsion stability, samples with and without antioxidants (control, 0.02% FSEY, 0.02% TBHQ) were evaluated at 25 °C and 60 °C.

**Figure 7:**
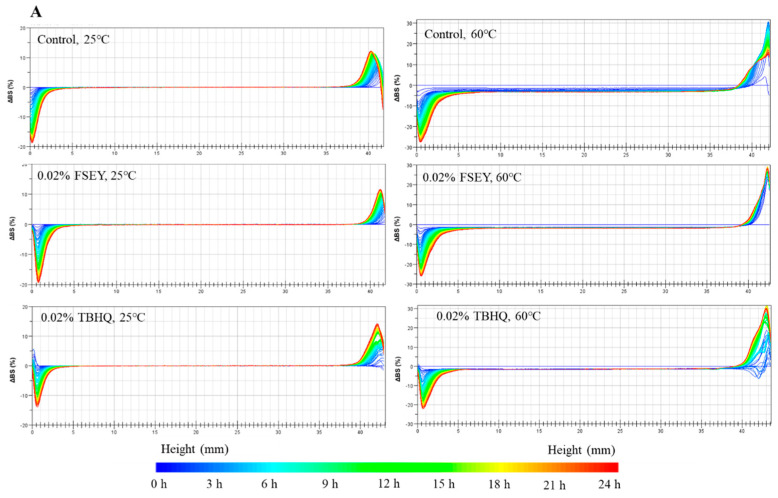

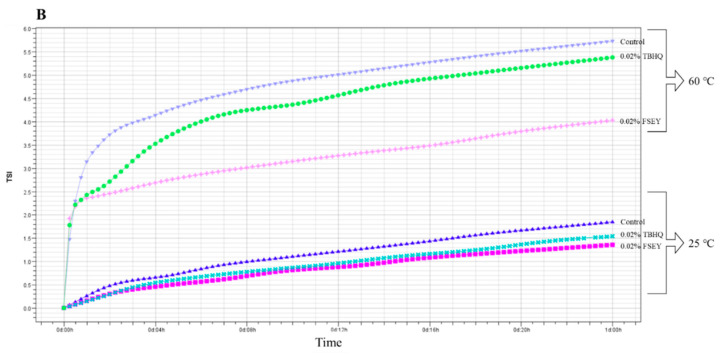
(**A**) Delta backscattering of oil-in-water emulsions, in the absence or in the presence of antioxidants, in different temperatures. (**B**) Turbiscan stability index of the oil-in-water emulsions. These data are represented as a function of time (0:00 to 24:00 h) and of sample height (0 to 42 mm).

In the Turbiscan analysis, the changes in backscattering (ΔBS) were calculated, taking the first scan in Figure 7A as a reference with a value of zero. When monitored at room temperature (25 °C), it can be seen that the ΔBS at the top of the emulsion increased slightly, while there was no significant difference between the three groups. In addition, when the scan temperature was increased to 60 °C, all the ΔBS values increased significantly, especially the control group. As the BS intensity is linked to the average diameter and number of particles [39], a high temperature resulted in an emulsion that was more unstable. Moreover, the backscattering intensity of the emulsions decreased in the bottom of the test tube due to the reduced concentration of droplets (clarification). In contrast, the increases at the top of the test tube were due to the droplet concentration increasing [38], and the double peaks that appeared in the top area represent demulsification. As shown in Figure 7A, at the top of the emulsion, a progressive increase in backscattering intensity was observed in all samples; in particular, double peaks appeared in the control sample. Emulsions without antioxidants exhibited serious demulsification phenomena, which were mostly observed at high temperatures. The demulsification phenomenon was not obvious in the FSEY and TBHQ groups. The figure also indicated a creaming phenomenon of the emulsion, which was caused by coalescence and flocculation. The ΔBS in the middle increased with an increasing particle size. From Figure 7A, a high temperature decreased the stability, and FSEY and TBHQ could inhibit the aggregation of oil droplets. These results are consistent with the particle size analysis and microscopy observation.

Many studies have used the ΔBS value to reflect emulsion instability. Wang et al. used the BS change rates to evaluate stabilizers, and they showed that smaller BS change rates reflect higher emulsion stability [38]. Ren et al. compared the ΔBS values at 20 °C and 80 °C. They concluded that heat can induce flocculation significantly [41]. Yang et al. analyzed changes in backscattering profiles within 3 h to evaluate the stabilities of flavonoid glycoside or quercetin-containing emulsions. They found that flavonoid glycoside could slow down the creaming and particle size variation processes of the emulsion system [42]. Similarly, as an antioxidant additive, FSEY presents a stronger ability to stabilize the emulsion than TBHQ.

These findings were also reflected in the TSI of the emulsions, which is shown in Figure 7B. Emulsions could undergo different destabilization processes over time. A higher value of TSI corresponded to a lower stability. Wang et al. reported that natural sesamol and sesamin enhanced the physical stability of emulsions used as antioxidants, with lower TSI values than control [43]. A comparison of the TSI values of the emulsions within 19 h was carried out by Wang et al. The TSI values decreased significantly when the PGPR (polyglycerol polyricinoleate) and casein gels were introduced into the aqueous phase [44]. The addition of fibrillated cellulose could also reduce Turbiscan stability index dose-dependently [41]. In our study, the TSI values of all groups increased continuously; especially the emulsion without any antioxidants. The stability of emulsion was enhanced with the addition of FSEY or TBHQ. Notably, a higher temperature decreased this stability, but FSEY could prevent droplet flocculation. It has been reported that surfactants or biopolymers could improve emulsion stability by providing steric hindrance to flocculation or coalescence [38]. FSEY is an oil-insoluble tetrapeptide and mainly distributed in the aqueous and interfacial phases. Here, the ability to improve emulsion stability may contribute to steric repulsion, while TBHQ is a water-insoluble additive and is mainly distributed in the oil and interfacial phases. Its steric hindrance is lower than that of FSEY. From this point of view, FSEY displayed a stronger stabilization of physical structure than TBHQ.

Collectively, the changes in the droplet size, zeta potential, microstructure, color parameters, ΔBS data and TSI of the emulsions suggest that heat decreased the stability of the emulsion, while the antioxidant FSEY could help to stabilize the emulsion. Although TBHQ was more effective in inhibiting lipid oxidation, we suggest FSEY as an additive in emulsions due to its better performance in retaining the physical stability of emulsion.

### 3.6. Further Antioxidant Activities of FSEY

**Figure 8:**
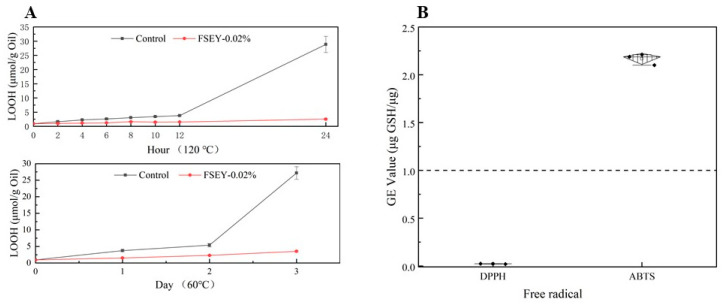
(**A**) Changes in primary lipid oxidation product values stored at 60 and 120 °C. (**B**) Capacities of FSEY in terms of scavenging DPPH and ABTS radicals. Values are expressed as mean ± SD (*n* = 3).

As mentioned above, FSEY demonstrated its capacity to inhibit lipid oxidation at 37 °C. Additionally, when incubated at 60 °C for 3 days and 120 °C for 24 h, this peptide antioxidant could also improve the oxidizing stability of O/W emulsions. Therefore, the results suggest that an elevated ambient temperature does not affect antioxidant activity (Figure 8A).

The ability to scavenge free radicals against ABTS and DPPH was analyzed. As shown in Figure 8B, FSEY demonstrated a lower DPPH-scavenging capability than GSH. Additionally, an excellent capacity against ABTS was observed, with a GE value of 2.20 ± 0.02. Zheng [45] suggested that Tyr and Tyr-containing peptides have a strong ABTS radical scavenging effect and non-DPPH radical scavenging activity, which is consistent with our results.

### 3.7. Prediction of the Toxicological Properties of FSEY

The carcinogenicity, mutagenicity, skin sensitization, and skin irritation of FSEY were predicted by the TOPKAT system. As shown in Table 3, FSEY showed no carcinogenicity or mutagenicity. Additionally, it was not a substance that exhibited potential for developmental toxicity. Although FSEY may be an eye irritant, there was no evidence of skin sensitization or skin irritation. Therefore, we believe FSEY is a highly safe additive.

## 4. Conclusions

In the current study, a hazelnut tetrapeptide, FSEY, was used as an antioxidant in an oil–water emulsion system. With final concentrations of 0.01%, 0.015%, and 0.02%, FSEY exhibited excellent antioxidant behaviors in the tested system. The presence of tetrapeptide-FSEY significantly reduced the primary and secondary oxidation products in the oil–water emulsion system, and reduced the generation of lipid free radicals. This peptide also achieved excellent antioxidant behaviors at high temperatures, and inhibited lipid oxidation by scavenging lipid free radicals; the Tyr- residue might be responsible for this activity.

Additionally, FSEY assisted in stabilizing the physical structure of the emulsion. In comparison with antioxidant-free emulsions, FSEY helped to inhibit the demulsification phenomenon caused by coalescence and flocculation, and it also stabilized the original color of emulsion. Additionally, TOPKAT demonstrated that it was a safe antioxidant. Therefore, FSEY may be an effective antioxidant additive to use in emulsion systems.

## Figures and Tables

**Table 1:** ζ-potential values (mV) and volume mean diameter.

	D [3,4] (μm)		Zeta Potential (mV)		pH	
	0 day	25 days	0 days	25 days	0 days	25 days
Control	0.52 ± 0.01 ^a^	0.84 ± 0.12 ^b^	−51.03 ± 1.03 ^a^	−55.01 ± 1.94 ^b^	7.03 ± 0.06 ^a^	5.33 ± 0.12 ^a^
FSEY	0.51 ± 0.00 ^a^	0.69 ± 0.15 ^ab^	−42.38 ± 1.58 ^b^	−46.51 ± 1.47 ^a^	7.02 ± 0.05 ^a^	7.10 ± 0.00 ^b^
TBHQ	0.51 ± 0.01 ^a^	0.59 ± 0.10 ^a^	−49.43 ± 3.34 ^a^	−50.09 ± 2.40 ^ab^	7.03 ± 0.02 ^a^	7.37 ± 0.06 ^c^

Different lower-case letters indicate significant differences between mean values for each treatment (*p* ≤ 0.05).

**Table 2:** Color changes of emulsions after storage at 37 °C.

Day	Sample	L	a	b	ΔE
0	Control	75.97 ± 0.37 ^c^	−0.50 ± 0.02 ^d^	−1.50 ± 0.04 ^c^	15.97 ± 0.36 ^ab^
	FSEY	76.10 ± 0.12 ^c^	−0.50 ± 0.01 ^d^	−1.51 ± 0.03 ^c^	15.84 ± 0.12 ^a^
	TBHQ	75.97 ± 0.21 ^c^	−0.49 ± 0.02 ^d^	−1.50 ± 0.02 ^c^	15.97 ± 0.20 ^ab^
14	Control	74.92 ± 0.24 ^c^	−0.61 ± 0.01 ^c^	−1.56 ± 0.03 ^bc^	16.02 ± 0.24 ^ab^
	FSEY	75.63 ± 0.04 ^c^	−0.67 ± 0.01 ^b^	−1.60 ± 0.04 ^b^	16.31 ± 0.03 ^b^
	TBHQ	74.70 ± 0.10 ^b^	−0.36 ± 0.03 ^e^	0.68 ± 0.06 ^e^	17.04 ± 0.10 ^c^
25	Control	73.45 ± 0.40 ^a^	−0.94 ± 0.01 ^a^	−2.08 ± 0.10 ^a^	18.55 ± 0.50 ^d^
	FSEY	75.97 ± 0.22 ^c^	−0.65 ± 0.00 ^b^	−1.26 ± 0.06 ^d^	15.93 ± 0.22 ^ab^
	TBHQ	73.52 ± 0.0 ^a^	0.60 ± 0.02 ^f^	2.90 ± 0.01 ^f^	18.36 ± 0.05 ^d^

Different lower-case letters indicate significant differences between mean values for each treatment (*p* ≤ 0.05).

**Table 3:** Prediction of toxicological properties.

Toxicological Properties	Model Prediction
Mutagenicity	Non-mutagen
Developmental toxicity potential (DTP)	Non-toxic
Ocular irritancy	Irritant
Skin irritancy	Non-irritant
Skin sensitization	Non-sensitizer
Rodent carcinogenicity (including NTP and FDA datasets; female or male)	Non-carcinogen

## Data Availability

Data are contained within the article and Supplementary Materials.

## Supplementary Materials

The following are available online at https://www.mdpi.com/article/10.3390/foods10061400/s1, Table S1: Bio-peptides from BIOPEP database with antioxidant activity against linoleic acid, Figure S1: Grand average of hydropathy (GRAVY) values of bio-peptides from BIOPEP database, Figure S2: Distribution of the droplets size obtained by software ImageJ (A for Conrtol-0 day, B for Control-25 day, C for FSEY-25 day, and D for TBHQ-25 day).

## References

[1] Kiokias S., Gordon M.H., Oreopoulou V. (2017). Effects of composition and processing variables on the oxidative stability of protein-based and oil-in-water food emulsions. Crit. Rev. Food Sci. Nutr..

[2] Sun Y., Wang W., Chen H., Li C. (2011). Autoxidation of unsaturated lipids in food emulsion. Crit. Rev. Food Sci. Nutr..

[3] Choe E. (2020). Roles and action mechanisms of herbs added to the emulsion on its lipid oxidation. Food Sci. Biotechnol..

[4] Ambrosone L., Mosca M., Ceglie A. (2007). Impact of edible surfactants on the oxidation of olive oil in water-in-oil emulsions. Food Hydrocoll..

[5] Decker E.A., McClements D.J., Bourlieu-Lacanal C., Durand E., Figueroa-Espinoza M.C., Lecomte J., Villeneuve P. (2017). Hurdles in predicting antioxidant efficacy in oil-in-water emulsions. Trends Food Sci. Technol..

[6] Ravera F., Dziza K., Santini E., Cristofolini L., Liggieri L. (2021). Emulsification and emulsion stability: The role of the interfacial properties. Adv. Colloid Interface Sci..

[7] Chen L., Ao F., Ge X., Shen W. (2020). Food-grade Pickering emulsions: Preparation, stabilization and applications. Molecules.

[8] Pei Y., Deng Q., McClements D.J., Li J., Li B. (2020). Impact of phytic acid on the physical and oxidative stability of protein-stabilized oil-in-water emulsions. Food Biophys..

[9] Celus M., Kyomugasho C., Keunen J., Loey A.M.V., Hendrickx M.E. (2019). Simultaneous use of low methylesterified citrus pectin and EDTA as antioxidants in linseed/sunflower oil-in-water emulsions. Food Hydrocoll..

[10] Li D., Zhao Y., Wang X., Tang H., Wu N., Wu F., Yu D., Elfalleh W. (2020). Effects of (+)-catechin on a rice bran protein oil-in-water emulsion: Droplet size, zeta-potential, emulsifying properties, and rheological behavior. Food Hydrocoll..

[11] Waraho T., McClements D.J., Decker E.A. (2011). Mechanisms of lipid oxidation in food dispersions. Trends Food Sci. Technol..

[12] Samdani G.K., McClements D.J., Decker E.A. (2018). Impact of phospholipids and tocopherols on the oxidative stability of soybean oil-in-water emulsions. J. Agric. Food Chem..

[13] García-Moreno P.J., Jacobsen C., Marcatili P., Gregersen S., Overgaard M.T., Andersen M.L., Sørensen A.-D.M., Hansen E.B. (2020). Emulsifying peptides from potato protein predicted by bioinformatics: Stabilization of fish oil-in-water emulsions. Food Hydrocoll..

[14] Pan X., Fang Y., Wang L., Shi Y., Xie M., Xia J., Pei F., Li P., Xiong W., Shen X. (2019). Covalent interaction between rice protein hydrolysates and chlorogenic acid: Improving the stability of oil-in-water emulsions. J. Agric. Food Chem..

[15] Shi Y., Liang R., Chen L., Liu H., Goff H.D., Ma J., Zhong F. (2019). The antioxidant mechanism of Maillard reaction products in oil-in-water emulsion system. Food Hydrocoll..

[16] García-Pérez P., Losada-Barreiro S., Bravo-Díaz C., Gallego P.P. (2020). Exploring the use of bryophyllum as natural source of bioactive compounds with antioxidant activity to prevent lipid oxidation of fish oil-in-water emulsions. Plants.

[17] O’Dwyer S.P., O’Beirne D., Eidhin D.N., O’Kennedy B.T. (2013). Effects of sodium caseinate concentration and storage conditions on the oxidative stability of oil-in-water emulsions. Food Chem..

[18] (2009). GB/T 24304-2009/ISO 6885:2006: Animal and Vegetable Fats and Oils-Determination of Anisidine Value.

[19] Jiang S., Xie Y., Li M., Guo Y., Cheng Y., Qian H., Yao W. (2020). Evaluation on the oxidative stability of edible oil by electron spin resonance spectroscopy. Food Chem..

[20] Cui N., Wang G., Ma Q., Zhao T., Li R., Liang L. (2020). Effect of cold-pressed on fatty acid profile, bioactive compounds and oil oxidation of hazelnut during oxidation process. LWT.

[21] Zhang X., Liu D., Jin T.Z., Chen W., He Q., Zou Z., Zhao H., Ye X., Guo M. (2021). Preparation and characterization of gellan gum-chitosan polyelectrolyte complex films with the incorporation of thyme essential oil nanoemulsion. Food Hydrocoll..

[22] Yang Q., Cai X., Yan A., Tian Y., Du M., Wang S. (2020). A specific antioxidant peptide: Its properties in controlling oxidation and possible action mechanism. Food Chem..

[23] Jie Y., Zhao H., Sun X., Lv X., Zhang Z., Zhang B. (2019). Isolation of antioxidative peptide from the protein hydrolysate of Caragana ambigua seeds and its mechanism for retarding lipid auto-oxidation. J. Agric. Food Chem..

[24] Faraji H., McClements D.J., Decker E.A. (2004). Role of continuous phase protein on the oxidative stability of fish oil-in-water emulsions. J. Agric. Food Chem..

[25] Gürbüz G., Kauntola V., Ramos Diaz J.M., Jouppila K., Heinonen M. (2018). Oxidative and physical stability of oil-in-water emulsions prepared with quinoa and amaranth proteins. Eur. Food Res. Technol..

[26] Djordjevic D., Cercaci L., Alamed J., McClements D.J., Decker E.A. (2007). Chemical and physical stability of citral and limonene in sodium dodecyl sulfate−chitosan and gum arabic-stabilized oil-in-water emulsions. J. Agric. Food Chem..

[27] Merkx D.W.H., Plankensteiner L., Yu Y., Wierenga P.A., Hennebelle M., Van Duynhoven J.P.M. (2021). Evaluation of PBN spin-trapped radicals as early markers of lipid oxidation in mayonnaise. Food Chem..

[28] Wu H. (2011). Structure-Activity Relationship of Antioxidative Peptides Derived from Whey Protein. Master’s Thesis.

[29] Shahidi F., Ambigaipalan P. (2015). Phenolics and polyphenolics in foods, beverages and spices: Antioxidant activity and health effects—A review. J. Funct. Foods.

[30] Wang M., Li C., Li H., Wu Z., Chen B., Lei Y., Shen Y. (2019). In Vitro and in silico antioxidant activity of novel peptides prepared from Paeonia ostii ‘Feng dan’ hydrolysate. Antioxidants.

[31] Saito K., Jin D.H., Ogawa T., Muramoto K., Hatakeyama E., Yasuhara T., Nokihara K. (2003). Antioxidative properties of tripeptide libraries prepared by the combinatorial chemistry. J. Agric. Food Chem..

[32] Porter W.L. (1980). Recent trends in food applications of antioxidants. Autoxidation in Food and Biological Systems.

[33] Joseph H. (2016). Designing Delivery Systems of Vitamin e to Enhance Its Stability and Bioaccessibility. Ph.D. Thesis.

[34] Yi J., Qiu M., Liu N., Tian L., Zhu X., Decker E.A., McClements D.J. (2020). Inhibition of lipid and protein oxidation in whey-protein-stabilized emulsions using a natural antioxidant: Black rice anthocyanins. J. Agric. Food Chem..

[35] Cheng C., Yu X., McClements D.J., Huang Q., Tang H., Yu K., Xiang X., Chen P., Wang X., Deng Q. (2019). Effect of flaxseed polyphenols on physical stability and oxidative stability of flaxseed oil-in-water nanoemulsions. Food Chem..

[36] Ng S., Khor Y., Lim H., Lai O.M., Wang Y., Wang Y., Cheong L.-Z., Imededdine N., Mansour L., Tan C. (2020). Fabrication of concentrated palm olein-based diacylglycerol oil–soybean oil blend oil-in-water emulsion: In-depth study of the rheological properties and storage stability. Foods.

[37] Chantrapornchai W., Clydesdale F., McClements D.J. (1999). Influence of droplet characteristics on the optical properties of colored oil-in-water emulsions. Colloids Surf. A Physicochem. Eng. Asp..

[38] Wang P., Chen C., Guo H., Zhang H., Yang Z., Ren F. (2018). Casein gel particles as novel soft Pickering stabilizers: The emulsifying property and packing behavior at the oil-water interface. Food Hydrocoll..

[39] Degrand L., Michon C., Bosc V. (2016). New insights into the study of the destabilization of oil-in-water emulsions with dextran sulfate provided by the use of light scattering methods. Food Hydrocoll..

[40] Raikos V. (2017). Encapsulation of vitamin E in edible orange oil-in-water emulsion beverages: Influence of heating temperature on physicochemical stability during chilled storage. Food Hydrocoll..

[41] Ren Y., Linter B.R., Foster T.J. (2020). Cellulose fibrillation and interaction with psyllium seed husk heteroxylan. Food Hydrocoll..

[42] Yang D., Wang X.-Y., Gan L.-J., Zhang H., Shin J.-A., Lee K.-T., Hong S.-T. (2015). Effects of flavonoid glycosides obtained from a Ginkgo biloba extract fraction on the physical and oxidative stabilities of oil-in-water emulsions prepared from a stripped structured lipid with a low omega-6 to omega-3 ratio. Food Chem..

[43] Wang X., Yu K., Cheng C., Yu X., McClements D.J., Huang W., Yang J., Huang F., Deng Q. (2019). Impact of sesame lignan on physical and oxidative stability of flaxseed oil-in-water emulsion. Oil Crop. Sci..

[44] Wang P., Cui N., Luo J., Zhang H., Guo H., Wen P., Ren F. (2016). Stable water-in-oil emulsions formulated with polyglycerol polyricinoleate and glucono-δ-lactone-induced casein gels. Food Hydrocoll..

[45] Zheng L. (2015). Structure-Activity Relationship and Directional Preparation of Antioxidant Peptide. Ph.D. Thesis.

