# Supplementary material for: Impact of Tetrapeptide-FSEY on Oxidative and Physical Stability of Hazelnut Oil-In-Water Emulsion
Source: Foods. 2021 Jun 17;10(6):1400. doi: 10.3390/foods10061400 (PMC8234661; doi:10.3390/foods10061400)
Supplement: Supplementary file 1 [file foods-10-01400-s001.zip › foods-1219186-supplementary.pdf]

foods

Supplementary material

Table S1. Bio-peptides from BIOPEP database with antioxidant activity against linoleic acid

ID no in BIOPEP database	Peptide sequence	GRAVY	ID no in BIOPEP database	Peptide sequence	GRAVY
3296	HHP	-2.67	3809	LQSGDALRVPSGTTYY	-0.37
3297	YHH	-2.57	3810	MQFHT	-0.54
3298	HHPL	-1.05	3811	PHCKRM	-1.47
3299	LHPH	-1.05	3826	LVNPHDHQN	-1.56
3300	PHH	-2.67	7867	GYY	-1.00
3301	HLH	-0.87	7945	YYY	-1.30
3302	LHH	-0.87	7946	YYG	-1.00
3303	HPLH	-1.05	7967	YGY	-1.00
3304	LLPHHH	-0.60	8253	LEELEEELEGCE	-0.92
3305	LH	0.30	8282	ISELGW	0.45
3306	HPHL	-1.05	8433	FLKPLFNAALKLLP	0.98
3307	PYY	-1.40	8439	YLMSR	-0.18
3308	HHLP	-1.05	8440	VLYEE	-0.06
3309	LPYY	-0.10	8441	MILMR	1.52
3310	LYPY	-0.10	8950	WCTSVS	0.58
3311	HPH	-2.67	8955	PYSFK	-0.96
3312	LLHH	0.30	8956	GFGPGL	0.63
3313	PLHH	-1.05	8957	VGGRP	-0.54
3314	LLPH	0.70	9370	VKRRGQDCIHGFCSD	-0.78
3315	HLHP	-1.05	9371	GQFNDKRWIPFG	-1.01
3316	HLPH	-1.05	9372	APIRMWYMYRKLTDMEPKPVA	-0.52
3317	HL	0.30	9450	LTEQESGVPVMK	-0.32
3318	LPHH	-1.05			
3319	HH	-3.20			
3320	HHPLL	-0.08			

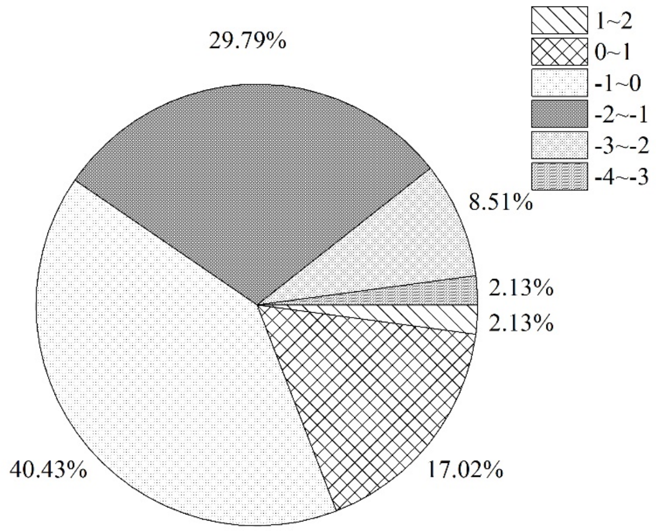

Figure S1. Grand average of hydropathy (GRAVY) values of bio-peptides from BIOPEP database.

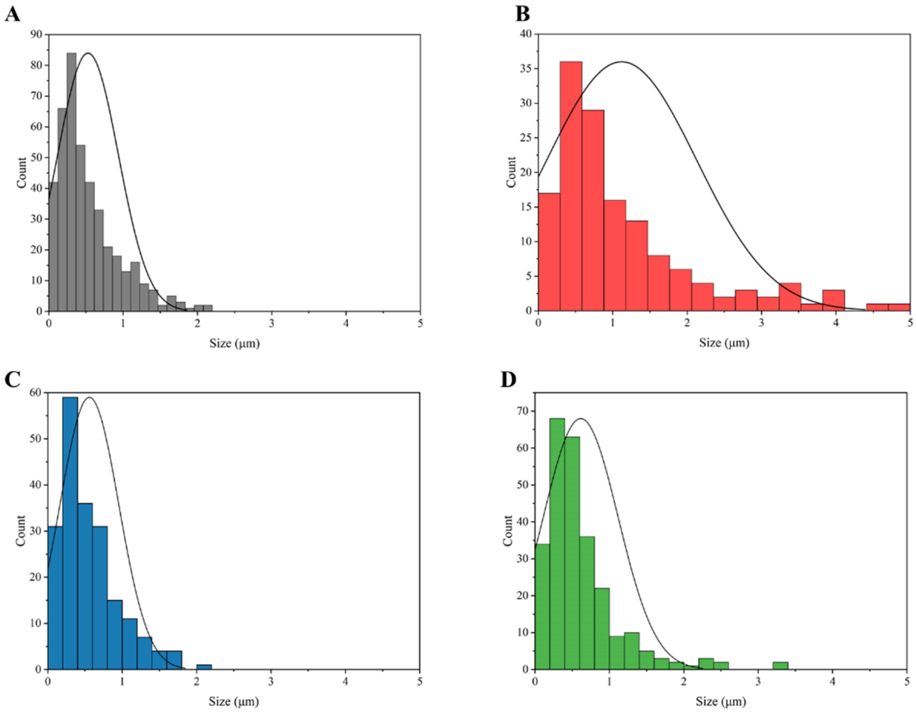

Figure S2. Distribution of the droplets size obtained by software ImageJ (A for Conrtol-0 day, B for Control-25 day, C for FSEY-25 day, and D for TBHQ-25 day).
